# Recurrent Guillain-Barré syndrome presenting as pharyngeal-cervical-brachial variant with three species of ganglioside antibodies:case report

**DOI:** 10.1186/s12883-022-02955-0

**Published:** 2022-11-28

**Authors:** Haihui Luan, Peng Zhang, Mingqing Zhen, Mei Li, Xiaowei Wang, Jianghua Xu

**Affiliations:** Department of Neurology, YEDA Hospital, 23-1 Economic and Technological Development Area, Yantai, Shandong China

**Keywords:** Guillain-Barré syndrome, Pharyngeal-cervical-brachial variant, Anti-ganglioside antibody, Recurrence

## Abstract

**Background:**

Guillain-Barré syndrome (GBS) is generally considered to be monophasic, and recurrent GBS (RGBS) is very rare. Pharyngeal-cervical-brachial (PCB) is a less common variant of GBS. There have been no cases reported describing RGBS showing different phenotype presenting as PCB variant with three species of ganglioside antibodies.

**Case presentation:**

We report a case of a 77-year-old female patient with GT1a, GD1a and sulfatide-seropositive PCB-GBS after prior episode of AMAN-GBS 13 years ago. Our patient showed oropharyngeal and cervicobrachial weakness associated with areflexia in the upper limbs and partially improved after 5 days of IVIG and physiotherapy.

**Conclusion:**

This study reports a rare case characterized as recurrent GBS after a long period, showing different phenotypes in different episodes with three different species of ganglioside antibodies. Further studies are required to obtain better understanding of RGBS and PCB variant.

## Background

Recurrent Guillain-Barré syndrome (GBS) is a rare condition that reportedly appears in 2–6% of patients with GBS [1, 2].The clinical and pathophysiological characteristics of RGBS have not been fully elucidated. The pharyngeal-cervical-brachial (PCB) variant, presents in 3% of patients with GBS [3–5]. Here we report a case of recurrent GBS showing different phenotype presenting as PCB with three species of ganglioside antibodies after prior episode 13 years ago.

## Case presentation

A 77-year-old female presented to the neurology department in our hospital with a 5-day history of dysarthria and upper limb weakness with steady progression. She suffered diarrhea 10 days previously and totally recovered before hospitalization. Further medical history of this patient revealed that this was her second occurrence of GBS. At the age of 64, she suffered the first attack, characterized by dysphagia, dysarthria and bilateral upper and lower limb weakness with areflexia. Weakness was more severe in the lower limbs(Medical Research Council [MRC] (grade 0/5)than the upper limbs(MRC grade 3/5). A Nerve conduction study(NCS) during her first presentation revealed axonal motor type polyneuropathy. She was diagnosed with acute motor axonal neuropathy(AMAN) according to Hadden’s criteria [6] and was treated with 5 days of IVIG and totally recovered. The medical record of the first episode was not available because she was admitted in another hospital in China and the records could not be shared. At this time, weakness was more severe in the upper limbs(MRC grade 2/5) than the lower limbs(MRC grade 4/5). Sensory disturbance and ataxia were not seen and the areflexia persisted. Routine laboratory examinations were normal, including complete blood count, blood chemistry, and immunological examinations. Brain magnetic resonance (MRI) found no abnormalities. NCS performed in another hospital 4days after neurological symptoms occurred revealed reduced motor conduction velocities and amplitudes in median, ulnar, tibial and peroneal nerves. The sensory nerves were normal and decreased F waves were detected in lower limbs. A lumbar puncture was performed 7days after clinical onset. The protein concentration in CSF was 567 mg/L with normal cellularity. A western blot analysis of anti-gangliosides(IgG anti-GM1, GM2, GM3, GM4, GD1a, GD1b, GT1a, GT1b, GQ1b,GD2,GD3,Sulfatide) was performed and GD1a positive(3+), GT1a positive(2+), Sulfatide(+) were found in serum, GD1a positive(3+) in CSF. In this case, the diagnosis of PCB variant, a different subtype from the initial diagnosis 13 years ago, was supported by acute and steadily progressive oropharyngeal, cervical, brachial weakness and mild lower limb weakness, antecedent infective symptoms, and positive for anti-GT1a antibody. Her symptoms were partially improved after the five doses of IVIG and her physical rehabilitation was initiated simultaneously. She did not suffer from any respiratory distress during her hospitalization and her vitals remained stable. She was discharged 10 days later and received regular physiotherapy for the next 3 months. At the 4-month follow-up, she had fully recovered.

## Discussion

The PCB variant of GBS is a rare condition which is characterized by rapidly progressive oropharyngeal and cervicobrachial weakness associated with hyporeflexia or areflexia in the upper limbs [5]. The strongest association for PCB is the presence of anti-GT1a antibody, which comes first with the identification of anti-GT1a and anti-GD1a antibodies in a patient with PCB weakness associated with mild leg weakness [7].

GBS is generally considered to be monophasic, but RGBS which is defined as two or more episodes of acute monophasic neuropathy with near or complete recovery between episodes [2], can occur in 2–6% of GBS [1, 2].In general, patients with RGBS and its variants show symptoms similar to previous episodes at relapse [8–10]. The heterogeneity of the phenotype is described in previous publications [11–14]. In these cases, most patients develop MFS or BBE, one with AMAN [15]. The long period between episodes of RGBS was also described in cases presented by Barbagallo [16]and Imam [17]. The coexistence of several antibody subtypes in patients with recurrent Guillain-Barré syndrome spectrum neuropathies was described in publications by Hwang [18] and Uchigami [19]. To our knowledge, there have been no cases describing RGBS presenting as PCB with anti-GD1a, GT1a and Sulfatide antibodies, 13 years after the first episode as AMAN-GBS. The antibody to GD1a is frequently elevated in patients with AMAN [20], anti-GT1a antibody is the culprit antibody of this episode and the role of the Sulfatide antibody is not clear currently. The most interesting feature of this case is the difference between two episodes apart from the rarity of recurring PCB-GBS itself. Previous case publications seem to suggest that the recurrence tends to be more severe than the initial episode in terms of clinical manifestations [9, 16, 18, 19], which was not seen in our case. The difference in clinical outcomes indicates that genetic and immunological host factors might affect the clinical manifestations and pathophysiology of RGBS.The management and treatment for GBS has been reviewed elsewhere [21], as for patients with pure PCB, close monitoring should continue since they are more likely to require intubation.

## Conclusion

The clinical manifestations of RGBS are heterogeneous, and the associated risk factors remain unclear. RGBS presenting as the PCB variant is very rare, yet its phenotype remains unfamiliar to many neurologists and general physicians. Our case report highlights the wide heterogeneity of the PCB variants and different phenotypes can occur in the same patient after one decade. Further studies are needed to obtain a better understanding of RGBS and PCB.

## Data Availability

The data used is available from the corresponding author on reasonable request.

## Abbreviations

PCB: Pharyngeal-cervical-brachial
IVIG: Intravenous immune globulin
MRI: Magnetic Resonance Imaging
MFS: Miller Fisher syndrome
BBE: Bickerstaff brainstem encephalitis

## References

[1] Wakerley BR, Uncini A, Yuki N (2014). Guillain-Barre and Miller Fisher syndromes–new diagnostic classification. Nat Rev Neurol.

[2] Kuitwaard K, van Koningsveld R, Ruts L, Jacobs BC, van Doorn PA (2009). Recurrent Guillain-Barré syndrome. J Neurol Neurosurg Psychiatry.

[3] Dimachkie MM, Barohn RJ (2013). Guillain-Barré syndrome and variants. Neurol Clin.

[4] Alter M (1990). The epidemiology of Guillain-Barré syndrome. Ann Neurol.

[5] Wakerley BR, Yuki N (2014). Pharyngeal-cervical-brachial variant of Guillain-Barre syndrome. J Neurol Neurosurg Psychiatry.

[6] Hadden RD, Cornblath DR, Hughes RA, Zielasek J, Hartung HP, Toyka KV (1998). Electrophysiological classification of Guillain-Barre syndrome: Clinical associations and outcome. Ann Neurol.

[7] Mizoguchi K, Hase A, Obi T (1994). Two species of antiganglioside antibodies in a patient with a pharyngeal-cervical-brachial variant of Guillain-Barré syndrome. J Neurol Neurosurg Psychiatry.

[8] Pyun SY, Jeong JH, Bae JS (2015). Recurrent Guillain-Barré syndrome presenting stereotypic manifestations, positive antiganglioside antibodies, and rapid recovery. Clin Neurol Neurosurg.

[9] Ito H, Hatanaka Y, Fukami Y (2018). Anti-ganglioside complex antibody profiles in a recurrent complicated case of GQ1b-seronegative Miller Fisher syndrome and Bickerstaff brainstem encephalitis: a case report. BMC Neurol.

[10] Heckmann JG, Dütsch M (2012). Recurrent miller fisher syndrome: clinical and laboratory features. Eur J Neurol.

[11] Mochizuki A, Ota K, Iijima M (1996). A case of relapsing Guillain-Barré syndrome following miller fisher syndrome. Rinsho Shinkeigaku.

[12] Hamaguchi T, Yamaguchi K, Komai K, Yamada M, Yokoji H, Satake R, Yoshino H (2003). Recurrent anti-GQ1b IgG antibody syndrome showing different phenotypes in different periods. J Neurol Neurosurg Psychiatry.

[13] Sharma V, Chan YC, Ong THL (2006). Bickerstaff’s brainstem encephalitis: can it recur?. J Clin Neurosci.

[14] Dong HQ, Liu Z, Tang Y, Lu Y, Wang Q, Jia JP (2011). Recurrent fisher-Bickerstaff syndrome: report of a Chinese case. Chin Med J (Engl).

[15] Jo YS, Choi JY, Chung H (2018). Recurrent Guillain-Barre Syndrome Following Urinary Tract Infection by Escherichia coli. J Korean Med Sci.

[16] Gaetano Barbagallo,Marcella Caggiula,Angela Lupo (2021). Recurrent Miller-Fisher syndrome overlapping Guillain-Barrè syndrome and Bickerstaff brainstem encephalitis: A case report. Clin Neurol Neurosurg.

[17] Kamran I, Liu A (2020). A case of Recurrent Guillain-Barre Syndrome observed by the same clinician 12 years apart. Clin Case Rep.

[18] Jihyeon Hwang,Ye Ji Kwon,Jong Kuk Kim (2019). Recurrent Guillain-Barré Syndrome with Anti-GT1a and Anti-GQ1b Ganglioside Antibodies. J Clin Neurol.

[19] Hirokazu  Uchigami, Yoshito Saito, Takeyuki Tsuchida (2021). Comparison of antibody reactivity in the first and second episodes of recurrent Miller Fisher syndrome. Neurol Sci.

[20] Yuki N, Hartung HP (2012). Guillain–Barré syndrome. N Engl J Med.

[21] Nortina Shahrizaila, Helmar C, Lehmann (2021). Satoshi Kuwabara. Guillain-Barré syndrome. The lancet.

